# The Use of ctDNA for *BRAF* Mutation Testing in Routine Clinical Practice in Patients with Advanced Melanoma

**DOI:** 10.3390/cancers14030777

**Published:** 2022-02-02

**Authors:** Paweł Sobczuk, Katarzyna Kozak, Sylwia Kopeć, Paweł Rogala, Tomasz Świtaj, Hanna Koseła-Paterczyk, Aleksandra Gos, Andrzej Tysarowski, Piotr Rutkowski

**Affiliations:** 1 Department of Soft Tissue/Bone Sarcoma and Melanoma, Maria Sklodowska-Curie National Research Institute of Oncology, 02-781 Warsaw, Poland; katarzyna.kozak@pib-nio.pl (K.K.); sylwia.kopec@pib-nio.pl (S.K.); pawel.rogala@pib-nio.pl (P.R.); Tomasz.switaj@pib-nio.pl (T.Ś.); hanna.kosela-paterczyk@pib-nio.pl (H.K.-P.); piotr.rutkowski@pib-nio.pl (P.R.); 2 Department of Experimental and Clinical Physiology, Laboratory of Centre for Preclinical Research, Medical University of Warsaw, 02-097 Warsaw, Poland; 3 Department Molecular and Translational Oncology, Maria Sklodowska-Curie National Research Institute of Oncology, 02-781 Warsaw, Poland; aleksandra.gos@pib-nio.pl (A.G.); andrzej.tysarowski@pib-nio.pl (A.T.); 4 Cancer Molecular and Genetic Diagnostics Department, Maria Sklodowska-Curie National Research Institute of Oncology, 02-781 Warsaw, Poland

**Keywords:** melanoma, liquid biopsy, ctDNA, *BRAF*, BRAF/MEK inhibitors

## Abstract

**Simple Summary:**

Assessment of *BRAF* mutation status is mandatory in advanced, previously untreated melanoma patients since it is present in 40–50% of cases and allows treatment with specific inhibitors. The testing is usually performed on the primary tumor or metastatic lesion; however, in some cases, liquid biopsy and analysis of circulating tumor DNA in the blood can be used. The aim of our study was to evaluate the clinical utility of plasma circulating tumor DNA analysis for *BRAF* mutation. We identified 46 patients (21 female, 25 male) who underwent such a procedure. A *BRAF* mutation was found in 45.7% of liquid biopsies and 44.8% of tissue samples. In 18 patients, therapy with BRAF/MEK inhibitors was initiated on the basis of the result of liquid biopsy. Our study confirms the clinical utility of *BRAF* mutation detection in liquid biopsy.

**Abstract:**

Assessment of *BRAF* mutation status is mandatory in advanced, treatment-naïve melanoma patients. Liquid biopsy can be an alternative in cases with inadequate or unavailable tumor tissue. The aim of our study was to evaluate the clinical utility of plasma circulating tumor DNA analysis for *BRAF* mutation testing and to assess outcomes of therapy with BRAF/MEK inhibitors initiated based on the liquid biopsy results. This was a retrospective single-center analysis of 46 patients (21 female, 25 male) with advanced melanoma who underwent circulating tumor DNA (ctDNA) *BRAF* mutation testing. A *BRAF* mutation was found in 45.7% (21/46) of liquid biopsies and 44.8% (13/29) of tissue samples. In patients with both ctDNA and tissue samples (*n* = 29), the concordance between the results of both tests was 82.8%. A *BRAF* mutation was detected in 7/17 (41.2%) patients with only ctDNA analysis. In 18 patients, therapy with BRAF/MEK inhibitors was initiated on the basis of the result of liquid biopsy. The objective response rate was 77.8 %, and the median PFS was 6.0 months. Our study confirms the clinical utility of *BRAF* mutation detection in plasma ctDNA. This study provides initial real-world data showing that treatment with BRAF/MEK inhibitors could be commenced based on liquid biopsy results.

## 1. Introduction

Precision medicine has revolutionized the diagnosis and treatment of many cancers, including melanoma. Historically, the survival of patients with advanced melanoma treated with chemotherapy was highly unsatisfactory. The discovery of the presence of *BRAF* gene mutations in melanoma cells has led to extensive research and development of selective BRAF inhibitors [1]. The first BRAF inhibitor, vemurafenib, was approved by the FDA and EMA in 2011 and 2012, respectively, based on the results of a pivotal phase III trial [2]. Later, the next generation of BRAF inhibitors, such as dabrafenib and encorafenib, was introduced into clinical practice [3,4]. Moreover, to improve efficacy and reduce resistance, MEK inhibitors cobimetinib, trametinib, and binimetinib were introduced [4,5,6]. Currently, the combination of BRAF and MEK inhibitors is a standard of care for the treatment of melanoma in advanced disease as well as adjuvant settings [7,8].

The presence of activating *BRAF* mutations in melanoma cells is a cornerstone of the antitumor activity of BRAF/MEK inhibitors. Around 40–50% of patients with cutaneous melanoma harbor such mutations located in codon 600 of the *BRAF* gene [1]. The most common variant is V600E (p.Val600Glu), but a number of other mutations such as V600K, V600D, V600R, V600M, and V600E2 have been described [9]. Testing for the presence of somatic *BRAF* mutations in melanoma is a crucial step in diagnosis and the qualification for treatment. The most commonly used method is the assessment of *BRAF* mutations in primary or metastatic tumor tissue, usually formalin-fixed paraffin-embedded (FFPE) specimens. However, such tissue samples can be difficult to obtain in some situations. Thus, new methods of testing are developed and introduced into clinical practice.

Liquid biopsies allow evaluating the presence of circulating tumor cells (CTCs) or circulating tumor DNA (ctDNA) in the blood or urine [10]. ctDNA can be isolated and sequenced to study the tumor genetic landscape. It has already been validated for testing for specific mutations that are characteristic of a variety of tumors, such as *BRAF* mutations in melanoma or EGFR in non-small cell lung cancer [11,12]. Despite the progress in the field of liquid biopsies, it is mostly used in clinical trials and research settings. Its availability in everyday clinical practice is becoming more common but remains very limited.

In our study, we aimed to analyze the clinical utility of ctDNA for *BRAF* mutation testing in patients with melanoma in routine clinical practice. We characterized patients in whom ctDNA testing was used, and their treatment outcomes with BRAF/MEK inhibitors if activating *BRAF* mutations were found in ctDNA.

## 2. Materials and Methods

We screened the electronic health records of patients with cutaneous melanoma treated at the Maria Skłodowska-Curie National Research Institute of Oncology in Warsaw to identify patients who underwent *BRAF* mutation testing from a liquid biopsy. We included patients with liquid biopsy performed between 1 January 2018 and 15 August 2020 to achieve at least a 12-month follow-up. All patients had undergone *BRAF* mutation testing by liquid biopsy, and tissue sample testing was also performed when possible. Collected data included basic demographic data, stage of the disease, times of requesting and obtaining the results of *BRAF* mutation testing from liquid biopsy and tissue sample if available, reasons for performing a liquid biopsy, and data about eventual treatment with BRAF inhibitors.

The study was approved by the local bioethics committee at the Maria Skłodowska-Curie National Research Institute of Oncology in Warsaw—opinion 13/2008.

Tests were performed on 4–5 mL plasma, and ctDNA was isolated immediately after collection. ctDNA was isolated from plasma with QIAamp^®^ Circulating Nucleic Acid Kit (QIAGEN, Hilden, Germany) and analyzed by quantitative polymerase chain reaction (qPCR) with EntroGen^®^ ctDNA BRAF Mutation Detection Kit or AmoyDx^®^ *BRAF* V600 Mutations Detection Kit (Amoy Diagnostics, Xiamen, China) according to the manufacturer’s protocols. These are CE-IVD-certified tests for molecular diagnostics in Europe and allow the detection of V600 mutations in the BRAF oncogene via mutation-specific amplification technology. According to the manufacturer, the EntroGen^®^ ctDNA BRAF Mutation test enables the detection of the V600E, V600E2, V600K, V600D, V600R, and V600M BRAF mutations, where the sensitivity varies depending on the variant, with the highest value of 0.05% of ctDNA with a mutant variant for the V600E mutation, and 1% for V600K. AmoyDx^®^ *BRAF* V600 Mutations Detection Kit enables the detection of the V600E, V600E2, V600K, V600R, V600D1, and V600D2 BRAF mutations in at least 1% of mutant DNA in a background of 99% of normal DNA. Neither test distinguishes the type of mutation in codon V600. The result was considered positive if the CT parameter was within the range specified in the manufacturer’s instructions. DNA from tissues was isolated with Qiagen QIAamp^®^ DNA Mini-Kit (QIAGEN) or Cobas^®^ DNA Sample Preparation Kit (Roche, Basel, Switzerland) and analyzed by therascreen^®^ *BRAF* RGQ PCR kit (QIAGEN), AmoyDx^®^ *BRAF* V600 Mutations Detection Kit (Amoy Diagnostics), or Cobas^®^ 4800 *BRAF* V600 Mutation Test (Roche). All tests detect activating *BRAF* mutations at codon 600. All analyses were performed in a laboratory certified by EMQN (European Molecular Genetics Quality Network, Manchester, UK) and GENQA (Genomic Quality Assessment, Edinburgh, UK).

Descriptive statistics were used to report the data and the Mann–Whitney U test for between-group comparisons. A Kaplan–Meier estimator was used to calculate survival data. The database was locked on 15 August 2021—patients alive at this time point were censored. Progression-free survival (PFS) was defined as the time between the start of the treatment and disease progression, death, or lost to follow-up, whichever occurred first. Overall survival (OS) was defined as the time from initiation of the therapy to death or lost to follow-up. The duration of response (DOR) was defined as the time interval between data of imaging when an objective response (OR), partial response (PR), or complete response (CR) was first noted and disease progression, death, or lost to follow-up, whichever occurred first. All patients treated with BRAF/MEK inhibitors were followed with computed therapy every 8–12 weeks to assess treatment response, which was evaluated according to RECIST 1.1 criteria. The turnaround time (TAT) for mutation testing was calculated as the time between ordering the test and obtaining the final results. PS Imago PRO 7.0 software was used for statistical analysis. A *p*-value of 0.05 was considered statistically significant.

## 3. Results

### 3.1. Patient Characteristics

We identified 46 patients (21 female, 25 male) diagnosed with cutaneous melanoma who underwent *BRAF* mutation testing from liquid biopsy (Table 1). A total of 93.6% of patients (*n* = 44) were of stage IV, and the remaining 6.4% (*n* = 2) were of stage III, at the time of the testing. A total of 78.3% (*n* = 36) of patients were treatment-naïve, and 34.8% of patients at the time of diagnosis had an LDH level ≥2xULN.

### 3.2. BRAF Mutation Testing

**Figure 1:**
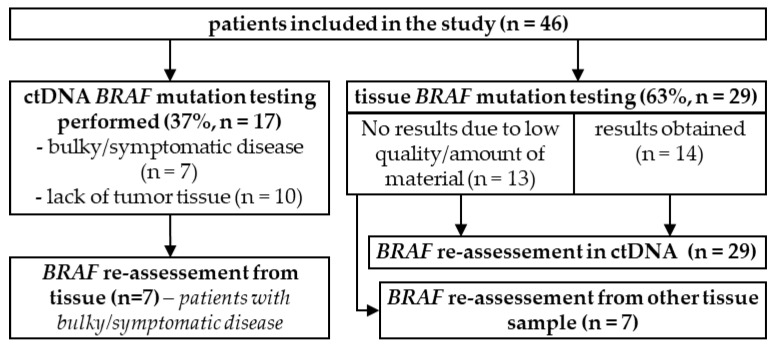
Flowchart of different *BRAF* mutation testing methods in the study population.

In 63% (*n* = 29) of patients, the *BRAF* mutation test was first ordered on tissue samples. In 13 of those 29 (44.8%) patients, a mutation assessment was not possible due to an insufficient amount or quality of samples; later, 6 of 13 patients had a complete reassessment from another tissue specimen. Seven patients required a quick verification of their *BRAF* status; thus, ctDNA analysis was performed while awaiting tissue sample results. For nine patients who were *BRAF* negative in a tissue sample, ctDNA evaluation was ordered to verify the results (Figure 1).

In 37% (*n* = 17) of patients, ctDNA assessment was ordered as the first diagnostic test—in 10 patients due to the lack of an available tissue sample, and in 7 to obtain results and start the treatment promptly. Further results from tissue samples were obtained later in all seven patients (Figure 1).

Overall, all 46 patients had *BRAF* mutation results from ctDNA, and 29 (63%) also had results from tumor tissue samples. Metastatic lymph nodes were the most common source of tested materials (55.2%, *n* = 16/29), followed by distant metastases (34.5%, *n* = 10/29) and primary tumors (10.3%, *n* = 3/29). The median turnaround time (TAT) for ctDNA testing results was only 1.07 days (range 0.2–6.0), while it was 9.2 days (2.9–38.0) for tissue testing (*p* < 0.001).

A *BRAF* mutation was detected in 45.7% (*n* = 21/46) of liquid biopsies and 44.8% (*n* = 13/29) of tissue samples. In patients with both ctDNA and tissue samples (*n* = 29), the agreement rate between both tests was 82.8%. In 84.6% of *BRAF* tissue-positive patients, A *BRAF* mutation was detected in ctDNA, while the lack of a mutation was confirmed in 81.3% of cases. The ctDNA testing sensitivity was 84.6%, and the specificity was 81.3% (Table 2). In patients who only had ctDNA analysis, a *BRAF* mutation was detected in 41.2% (*n* = 7/17).

### 3.3. BRAF/MEK Inhibitor Treatment

**Figure 2:**
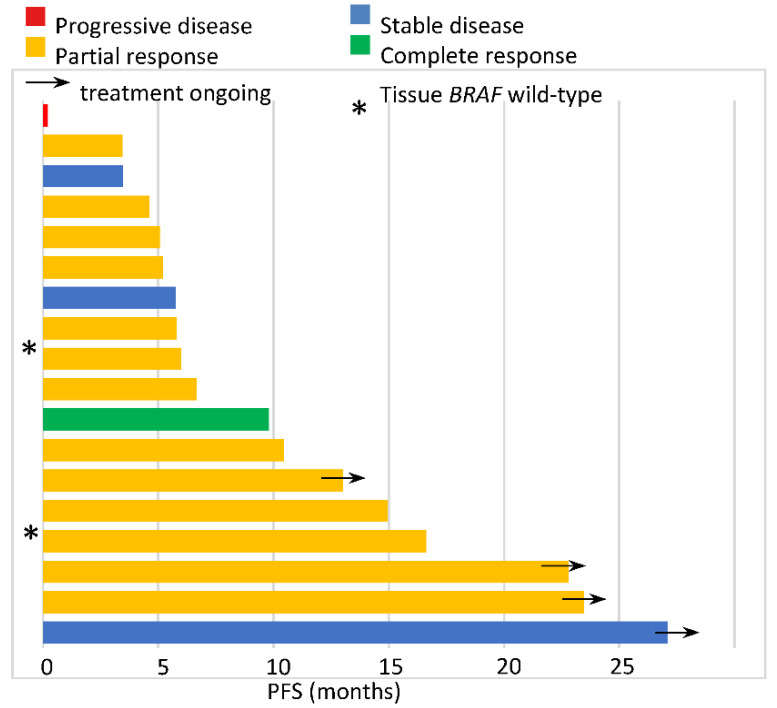
Progression-free survival with BRAF/MEK inhibitors administered based on the ctDNA analysis.

Overall, 18 patients were treated with BRAF/MEK inhibitors in the first line based on the ctDNA *BRAF* mutation testing. The median PFS was 6.0 months (95%CI 4.1–7.8), and treatment is ongoing in two patients (Figure 2). The ORR was 77.8% with 1 complete response (CR; 5.6%) and 13 partial responses (PR; 72.2%). The disease control rate (DCR) was 94.4%—only one patient experienced disease progression as the best response. The median DOR was 4.0 months (95% CI 0–10.3). Six patients died, and the median OS was 12.8 months (95% CI 0–25.8).

Two of three patients with *BRAF*-positive ctDNA but negative tissue *BRAF* (all determined by the Cobas 4800 BRAF V600 Mutation Test) were treated with BRAF/MEK inhibitors, both achieving a PR (Figure 2). The PFS was 15.0 and 5.8 months, while the duration of response was 10.4 and 3.9 months. One of two patients with *BRAF*-negative ctDNA but a positive tissue *BRAF* status received BRAF inhibitors with stable disease for 27.1 months and are continuing treatment (Figure 2).

Five patients received BRAF/MEK inhibitors solely based on the ctDNA analysis (no tissue results obtained), with one achieving a complete response, three partial responses, and one stabilization of the disease. The PFS in these patients was 26.1, 19.2, 9.8, 6.0, and 3.5 months.

## 4. Discussion

Testing for the presence of *BRAF* V600 mutations is a standard procedure when commencing the treatment of patients with melanoma. It is preferably performed on a tissue sample. However, in some cases, the quantity or quality of material can be inappropriate and alternative methods, such as liquid biopsies, are necessary. Here, we reported a retrospective analysis of all consecutive liquid biopsies performed in a tertiary center outside clinical trials.

Previous studies have shown high concordance in detecting somatic mutations in ctDNA and melanoma tumor samples. On average, 70–90% of single-nucleotide variants were found both in the plasma and tissue [13]. Based on this observation, ctDNA testing has become more common in patients with melanoma. Numerous previous studies reported a plasma ctDNA sensitivity of 50–100% and 75–100% specificity in detecting *BRAF* mutations [12,14,15,16,17,18]. Due to the study’s design, we were not able to determine the sensitivity and specificity. Although we observed over 80% concordance between both assays, which is in line with previously reported sensitivity and specificity rates, it has to be noted that only 2/3 of patients had both ctDNA and tissue samples analyzed, which could impact the results. Moreover, the majority of previous studies analyzed data obtained from clinical trials, not routine clinical practice.

Various technologies are used for the detection and quantification of plasma ctDNA and the detection of specific mutations. Standard qPCR, used in this study, detects mutations if present in >1% of ctDNA, while novel techniques such as digital PCR, digital droplet PCR, or BEAming can detect mutant and wild-type DNA at ratios greater than 0.01% [11,18]. A higher sensitivity of ctDNA tests is reported in patients with stage IV compared to those with stage III melanoma [19]. Additionally, the location of metastases can affect the detectability of ctDNA—patients with visceral, bone, or lymph node metastases tend to display higher levels of ctDNA as compared with patients with brain metastases or subcutaneous involvement [20].

It has been proven that higher levels of detectable ctDNA correlate with worse melanoma-specific and distant metastasis recurrence-free survival in patients with stage III melanoma, lower response rates, and worse PFS and OS in patients with stage IV melanoma [14,17,18,21,22,23,24]. Moreover, the response to BRAF/MEK inhibitors correlates with a diminishing amount of ctDNA with *BRAF* mutations, while rebounding levels of circulating *BRAF* V600E are observed at the time of disease progression [14,24]. The increase in the ctDNA level can precede the detection of relapses by imaging or clinical evaluation [12]. In our study, ctDNA was used only to determine *BRAF* mutational status, and quantitative analysis was not performed, which is one of the study limitations. Nevertheless, detecting *BRAF* mutations in liquid biopsies enabled the initiation of BRAF/MEK inhibitor therapy in 18 patients. All of these patients were treated with BRAF/MEK inhibitors in the first-line setting, which best reflects the disease burden in these patients. Currently, the preferred upfront treatment for metastatic melanoma is immunotherapy, as it provides the most durable outcomes. Updated results from phase III randomized clinical trials demonstrated 5-year overall survival rates of 39–44% for anti-PD1 monotherapy and 52% for the nivolumab plus ipilimumab combination [25,26]. In comparison, 5-year OS rates in patients treated in phase III trials with dabrafenib plus trametinib and vemurafenib plus cobimetinib were 34% and 31%, respectively [27,28]. Recently published data from two randomized trials (SECOMBIT and DREAMseq) evaluating the optimal sequencing of *BRAF*-targeted therapy and immune checkpoint inhibitor therapy showed a survival advantage of the combination of nivolumab plus ipilimumab over first-line targeted therapy [29,30]. However, in cases where the disease is symptomatic and rapidly progressing, and the patient’s condition appears to be deteriorating, *BRAF*-targeted therapy is still the preferred frontline treatment. In our study, we observed a median PFS of 6.0 months and an ORR in 77.8 % of patients. This is shorter than in the pivotal phase III trials, but similar to real-life evidence [31]. It is also possible that patients treated in real-life settings and in this study could have more unfavorable prognostic factors. These efficacy data are consistent with results from randomized controlled trials, which showed that the median PFS in patients with an elevated LDH level or higher disease burden ranged from 5.5 to 9.2 months [32]. The unquestionable advantage of liquid biopsy in patients with symptomatic disease is the short turnaround time, which allows a prompt initiation of systemic therapy.

ctDNA testing also offers an attractive alternative to tumor tissue profiling in cases where the tissue is inaccessible or inadequate for molecular analysis, or when the patient’s condition does not allow an invasive tissue biopsy. In our study, all the patients that received BRAF/MEK inhibitors solely based on the ctDNA analysis derived a clinical benefit, including one patient achieving a complete response.

Of note, our analysis also included *BRAF* V600 plasma-positive/tissue-negative patients. In these three patients, *BRAF* mutation status analysis in tumor tissue was performed using the Cobas 4800 *BRAF* V600 Mutation Test. This could be explained by findings from previous studies which showed that the Cobas test was less sensitive in detecting mutations other than the single-nucleotide V600E mutation [33,34]. However, due to the characteristics of the used diagnostic assays, we were not able to verify a specific type of detected V600 mutation. Two of these three patients were treated with BRAF/MEK inhibitors, with a partial response observed in both patients. Taken together, the findings of this study indicate that plasma ctDNA testing may serve as an important diagnostic tool to help ensure optimal treatment for melanoma patients.

Several study limitations have to be mentioned. This is a retrospective analysis of patients diagnosed and treated in routine clinical practice, performed in a small cohort of patients. ctDNA analysis was applied only in a selected population of patients in whom tissue testing was not possible or who required prompt commencement of treatment due to bulky or symptomatic disease. Moreover, as mentioned above, the assays used in diagnostic procedures do not distinguish the type of mutation in codon V600, and levels of mutated ctDNA were not measured; thus, assessing correlations with treatment outcomes was not possible.

## 5. Conclusions

We have shown that ctDNA is a feasible source of genetic material for *BRAF* mutation assessment in clinical practice in advanced melanoma patients, especially when a tissue sample is not available or in patients with rapidly progressive disease. The over 80% concordance between the results obtained from tissue and ctDNA highlights the clinical utility of liquid biopsies. Their minimal invasiveness, low cost, reproducibility, and short time required to obtain the results are the main advantages of liquid biopsies. Moreover, they allow overcoming limitations such as the availability and quality of tissue material and tumor heterogeneity.

This study provides initial real-world data showing that treatment with BRAF/MEK inhibitors could be commenced based on the results of liquid biopsy.

## Figures and Tables

**Table 1:** Study population characteristics.

Characteristics		*n* (%)
Gender	Male	21
	Female	25
Stage	III	2 (6.4)
	IV	44 (93.6)
Previous treatment	Yes	10 (21.7)
	*Anti-PD-1*	*9 (90.0)*
	*chemotherapy*	*1 (10.0)*
LDH	Normal	13 (28.3)
	ULN < 2xULN	11 (23.9)
	≥2xULN	16 (34.8)
	Not available	6 (13.0)
Organs with metastatic lesions	>3	11 (23.9)
Brain metastases		15 (32.8)

LDH—lactate dehydrogenase; ULN—upper level of normal.

**Table 2:** Number of patients with *BRAF*-mutated wild-type melanoma according to the diagnostic modality and concordance of results between ctDNA and tissue sample testing.

Patients with Both ctDNA and Tissue Tests (*n* = 29)		ctDNA *BRAF*	
		Mutated	Wild-Type
Tissue *BRAF*	Mutated	11	2
	Wild type	3	13
		Concordance between assays’ results, 82.8%	

## Data Availability

The data presented in this study are available on request from the corresponding author. The data are not publicly available due to inclusion of sensitive genetic information.
